# Structure of tyrosine aminotransferase from *Leishmania infantum*

**DOI:** 10.1107/S2053230X14007845

**Published:** 2014-04-25

**Authors:** M. A. Moreno, A. Abramov, J. Abendroth, A. Alonso, S. Zhang, P. J. Alcolea, T. Edwards, D. Lorimer, P. J. Myler, V. Larraga

**Affiliations:** a Departamento de Microbiología Molecular, Centro de Investigaciones Biológicas, Consejo Superior de Investigaciones Científicas (CSIC), Calle Ramiro de Maeztu 9, 28040 Madrid, Spain; b Seattle Structural Genomics Center for Infectious Disease (SSGCID), USA; c Seattle Biomedical Research Institute, 307 Westlake Avenue North, Seattle, WA 98109, USA; d Emerald Bio Inc., 7869 NE Day Road West, Bainbridge Island, WA 98110, USA; e Department of Global Health and Department of Biomedical Informatics and Medical Education, University of Washington, Seattle, WA 98125, USA

**Keywords:** tyrosine aminotransferase, *Leishmania infantum*

## Abstract

The trypanosomatid parasite *Leishmania infantum* is the causative agent of visceral leishmaniasis (VL), which is usually fatal unless treated. VL has an incidence of 0.5 million cases every year and is an important opportunistic co-infection in HIV/AIDS. Tyrosine aminotransferase (TAT) has an important role in the metabolism of trypanosomatids, catalyzing the first step in the degradation pathway of aromatic amino acids, which are ultimately converted into their corresponding l-2-oxoacids. Unlike the enzyme in *Trypanosoma cruzi* and mammals, *L. infantum* TAT (LiTAT) is not able to transaminate ketoglutarate. Here, the structure of LiTAT at 2.35 Å resolution is reported, and it is confirmed that the presence of two *Leishmania*-specific residues (Gln55 and Asn58) explains, at least in part, this specific reactivity. The difference in substrate specificity between leishmanial and mammalian TAT and the importance of this enzyme in parasite metabolism suggest that it may be a useful target in the development of new drugs against leishmaniasis.

## Abstract 2

The structure of the tyrosine aminotransferase from the parasitic protozoa *L. infantum* was solved to 2.35 Å resolution. The difference in substrate specificity and enzymatic activity between leishmanial and mammalian TAT is explained based on the presence of two residues (Gln55 and Asn58).

## 1. Introduction   

The leishmaniases are a group of vector-borne parasitic diseases that threaten about 350 million people in 88 countries around the world, mostly in developing countries. *Leishmania infantum* (family Trypanosomatidae) is the causative agent of zoonotic visceral leishmaniasis (VL) in the Mediterranean basin, where dogs are the main reservoir. *L. infantum* is an important opportunistic human parasite, resulting in increasing co-infection with HIV (Cruz *et al.*, 2006 ▶). A major outbreak of VL in humans has recently been reported in central Spain (Arce *et al.*, 2013 ▶).

*L. infantum* has a digenetic biological life cycle, alternating between a mobile extracellular promastigote in the insect vector and an immobile intracellular amastigote in the mammalian host (Handman, 2001 ▶). Inside the gut of the sand-fly vector, an environment rich in proteins and amino acids (Rosenzweig *et al.*, 2008 ▶), promastigotes undergo metacyclogenesis to increase their infectivity, and differentiate into amastigotes once inside the phagolysosome of mammalian phagocytes, where the availability of these nutrients is limited (McConville *et al.*, 2007 ▶). Under these conditions, amino-acid catabolism becomes an important source of energy for the parasite by cytosolic NADH re-oxidation (Nowicki & Cazzulo, 2008 ▶) and methionine recycling (Berger *et al.*, 2001 ▶; Berger *et al.*, 1996 ▶).

Aromatic amino-acid catabolism is highly active in trypanosomatids, but degradation to CO_2_ and water is not complete as in mammals. This pathway consists of two steps leading to a reduced l-2-hydroxyacid product whose excretion has been correlated with virulence (Montemartini *et al.*, 1994 ▶). The first step of this pathway is carried out by a tyrosine aminotransferase (TAT) belonging to the fold type I aminotransferases within the pyridoxal phosphate (PLP)-dependent superfamily (Jensen & Gu, 1996 ▶). One of the most studied enzymes belonging to this fold group is the broad substrate-specificity tyrosine aminotransferase from *Trypanosoma cruzi* (TcTAT), the structure of which has recently been resolved (Blankenfeldt *et al.*, 1999 ▶; Montemartini *et al.*, 1993 ▶, 1995 ▶).

However, the biological function of TAT in trypanosomatids is not yet clear, although previous studies detected an increase in transcript abundance of TAT in infective promastigotes of *L. infantum* (Alcolea *et al.*, 2009 ▶) and in a benznidazole-resistant *T. cruzi* strain (Rego *et al.*, 2008 ▶). These data suggest that TAT in trypanosomatids may play an important role in the infectivity of the parasite and its resistance against chemotherapy. We have solved the structure of *L. infantum* tyrosine aminotransferase (LiTAT) covalently bound to PLP, providing a structural basis for its enzymatic activity. The difference in substrate specificity between leishmanial and mammalian TAT and the importance of this enzyme in parasite metabolism suggest that it may be a promising target in the development of new drugs against leishmaniasis.

## 2. Methods   

### 2.1. Protein expression and purification   

The full-length LiTAT (*LinJ.36.2490*; NCBI XP_001469829.1) gene encoding a protein of 448 amino acids (UniProt A4IDL0) was PCR-amplified from *L. infantum* (MHOM/ES/98/10445) genomic DNA using oligonucleotides containing *Bam*HI and *Hin*dIII restriction sites (LiTAT_Fw, 5′-ACGGGATCCACGATTGATACGCAGGCC-3′, and LiTAT_Rv, 5′-ACGAAGCTTCTACTTCTTGTGGCGCTC­GC-3′). A truncated version of the gene (LiTAT_truncated) lacking the first 114 nucleotides of the annotated sequence was also amplified using oligonucleotides containing the same restriction sites (LiTAT_truncated_Fw, 5′-ACGGGATCCACGAGTTTTCGCCGT­ATCGC-3′, and LiTAT_Rv). LiTAT and LiTAT_truncated were cloned into the pRSET-A vector (Invitrogen) and recombinant proteins were expressed in *Escherichia coli* BL21 (DE3) pLysS. Cells were grown in 3 l LB medium at 310 K until the OD_600_ reached 0.5. Isopropyl β-d-1-thiogalactopyranoside (IPTG) was then added to a final concentration of 1 m*M* and induction continued for 2 h at 298 K, after which the harvested cells were frozen at 193 K. The frozen cell pellet was thawed and resuspended by vortexing in 200 ml lysis buffer [20 m*M* Tris–HCl pH 7.9, 500 m*M* NaCl, 4 µ*M* PLP, 50 m*M* imidazole, 0.05 mg ml^−1^ lysozyme and protease-inhibitor cocktail (Roche, Basel, Switzerland; used following the manufacturer’s instructions)]. The cell suspensions were lysed by sonication for 15 min and clarified by centrifugation on a Sorvall SS-34 at 27 000*g*. The clarified solutions were syringe-filtered through a 0.45 µm filter. The proteins were purified at room temperature by immobilized metal-affinity chromatography on a HisTrap FF 5 ml column (GE Healthcare) equilibrated with binding buffer (20 m*M* Tris–HCl pH 7.9, 500 m*M* NaCl, 4 µ*M* PLP, 50 m*M* imidazole). The proteins were eluted with eight column volumes of elution buffer (20 m*M* Tris–HCl pH 7.9, 500 m*M* NaCl, 4 µ*M* pyridoxal phosphate, 500 m*M* imidazole). Size-exclusion chromatography was performed at room temperature using a HiLoad 26/60 Superdex 75 prep-grade column (GE Healthcare, Piscataway, New Jersey, USA) equilibrated in SEC buffer [20 m*M* HEPES pH 7.0, 300 m*M* NaCl, 5%(*v*/*v*) glycerol, 1 m*M* tris(2-carboxyethyl)­phosphine hydrochloride]. Both proteins eluted as single peaks which correspond to the dimeric form in solution based on the estimated molecular weight. The calibration curve obtained using the Low Molecular Weight Kit (GE Healthcare) allowed determination of the molecular weights of both proteins once the gel base distribution coefficient value (*K*_av_) has been calculated from the measured elution volume (Supplementary Fig. S1^1^). Pooled fractions were concentrated at 277 K using an Amicon Ultra-15 30 kDa molecular-weight cutoff concentrator (Millipore, Billerica, Massachussets, USA) to 64.6 mg ml^−1^ for LiTAT and 51.6 mg ml^−1^ for LiTAT_truncated. The purity of both proteins was assessed to be >95% by SDS–PAGE.

### 2.2. Crystallization and structure solution   

Purified LiTAT and LiTAT_truncated proteins were used for crystallization screening at 21.0 and 21.3 mg ml^−1^, respectively, using four sparse-matrix screens: JCSG+, MCSG1 (Emerald Bio), Morpheus and PACT (Molecular Dimensions). Hexagonal crystals were obtained under several conditions, but few diffracted well. Crystals of LiTAT_truncated from Morpheus screen condition B11 (10% PEG 4000, 20% glycerol, 30 m*M* NaF, 30 m*M* NaBr, 30 m*M* NaI, 100 m*M* Bicine/Tris–HCl pH 8.5) were vitrified by plunging them into liquid nitrogen. X-ray diffraction data were collected on LS-CAT beamline 21-ID-F at the Advanced Photon Source, Argonne National Laboratory at a temperature of 100 K using a Rayonix MX-225 detector.

The diffraction data were reduced with the *XDS* suite (Kabsch, 2010 ▶) to 2.35 Å resolution (Table 1 ▶). Molecular replacement was performed with *Phaser* (McCoy *et al.*, 2007 ▶) using the structure of TcTAT (PDB entry 1bw0; Blankenfeldt *et al.*, 1999 ▶) as the search model. Molecular-replacement phases were improved including twofold NCS averaging with *Parrot* (Cowtan, 2010 ▶). An initial model was then built using *Buccaneer* (Cowtan, 2006 ▶). The model was then improved using iterative cycles of manual model building in *Coot* (Emsley *et al.*, 2010 ▶) and refinement with *phenix.refine* (Adams *et al.*, 2010 ▶). The resolution cutoff was *I*/σ(*I*) > 2 for the highest shell.

Structure factors and coordinates have been deposited in the PDB as entry 4ix8.

### 2.3. Determination of the activity of LiTAT_truncated   

The activity of LiTAT_truncated was assayed by the method of Diamondstone (1966 ▶) without the addition of diethylthiocarbamate. One unit of enzyme activity is defined as the amount of LiTAT that catalyzes the formation of 1 µmol *p*-hydroxyphenylpyruvate (measured as *p*-hydroxybenzaldehyde). The final enzymatic activity values are the means of four determinations.

## 3. Results and discussion   

### 3.1. Comparison of LiTAT with other closely related aminotransferases   

The primary sequence of the TAT protein is highly conserved among all *Leishmania* spp., while orthologues annotated in other trypanosomatids, such as *T. cruzi*, *T. rangeli* and *Crithidia acanthocephali*, show <50% sequence identity to the *Leishmania* sequence. *L. infantum* contains only a single copy of the TAT gene (*LinJ.36.2490*), while in *T. cruzi* there are around 70 copies of the TcTAT gene (Bontempi *et al.*, 1993 ▶). Interestingly, the TAT gene appears to be absent from the African trypanosomes (such as *T. brucei*), which do not have an intracellular immobile stage where the availability of nutrients is limited. Sequence alignment (Supplementary Fig. S2) shows a low degree of sequence identity between LiTAT and mammalian liver TAT (37%) and also between LiTAT and TcTAT (44%). Interestingly, the N-terminal 38 amino acids of mammalian liver TAT which were previously proposed to be involved in the rapid degradation of the protein (Hargrove *et al.*, 1989 ▶) are present in LiTAT (albeit with little sequence conservation) and are absent from the *T. cruzi* enzyme.

### 3.2. Crystal structure of LiTAT   

The N-terminal domain was too disordered to be modelled in full-length LiTAT, but its deletion in LiTAT_truncated did not affect the overall structure of the protein. Indeed, the best resolution (2.35 Å) was achieved for the LiTAT_truncated PLP-bound structure. The final model has a crystallographic *R* value of 17.3% and an *R*_free_ value of 20.3% (Table 1 ▶). The asymmetric unit of the crystal in space group *P*3_2_21 is formed by two identical polypeptide chains (*A* and *B*), forming a homodimer that is also present in solution (see Supplementary Fig. S1). For chain *A* residues Ser40–Lys448 could be built, while for chain *B* only residues Ser40–Ile439 could be built. However, several loop regions (Asp62–Asn63 in chain *A* and Asp62–Ser72, Glu363–Gly367, Lys382–Ser393 and Glu404–Glu405 in chain *B*) were too disordered to be modelled.

The five closest structural homologues of LiTAT were identified using the *DaliLite* v.3 server (Holm & Rosenström, 2010 ▶): TcTAT (PDB entry 1bw0; *Z*-score 59.2, 44% identity; Blankenfeldt *et al.*, 1999 ▶), TAT from *Homo sapiens* (PDB entry 3dyd; *Z*-score 54.4, 37% identity; Structural Genomics Consortium, unpublished work), alanine aminotransferase from *Pyrococcus furiosus* (PDB entry 1xi9; *Z*-score 53.1, 28% identity; Southeast Collaboratory for Structural Genomics, unpublished work), TAT from *Mus musculus* (PDB entry 3pdx; *Z*-score 52.8, 38% identity; Mehere *et al.*, 2010 ▶) and α-aminotransferase from *P. horikoshii* (PDB entry 1gd9; *Z*-score 48.4, 22% identity; Ura *et al.*, 2001 ▶). These alignments show the low percentage similarity between LiTAT and the other closely related orthologues.

**Figure 1:**
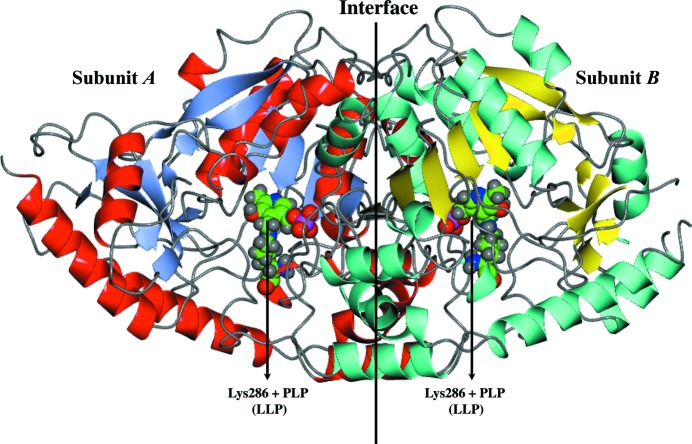
Dimeric crystal structure of LiTAT solved at 2.35 Å resolution. β-Sheets and α-­helices are shown as ice-blue and red ribbons for subunit *A* and as cyan and yellow ribbons for subunit *B*, respectively. The PLP molecule bound to Lys286 in each subunit is highlighted as a sphere model with C atoms in green.

The structure of each LiTAT subunit shows the typical fold type I of the aminotransferases (McPhalen *et al.*, 1992 ▶), with each monomer having two domains (Fig. 1 ▶). The larger of these domains (Asp91–Arg339) forms an internal core of seven sheets with β2 antiparallel to the rest. The core of the β-sheets is enclosed by α-helices. The smaller discontinuous domain comprises residues from the N-terminus (Lys65–Pro90) and C-terminus (Thr340–Lys448) which are involved in substrate recognition. As in TcTAT, the N-terminal residues (Ser40–Ser47) of LiTAT_truncated are involved in interaction between subunits (Blankenfeldt *et al.*, 1999 ▶).

LiTAT contains 13 cysteine residues per subunit; however, there are no disulfide bonds. Thus, it appears that the disulfide bond-mediated inactivation observed for mammalian TATs (Mehere *et al.*, 2010 ▶) is not used in LiTAT.

### 3.3. Substrate recognition   

**Figure 2:**
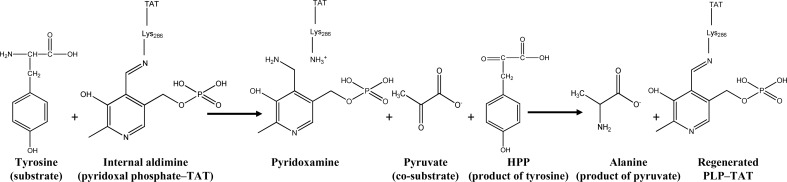
Transamination in two steps between tyrosine as the amino donor and pyruvate as the amino acceptor catalyzed by LiTAT. The end products of the reaction are alanine and *p*-hydroxyphenylpyruvate (HPP); the latter is reduced to *p*-hydroxyphenyllactate by a dehydrogenase in trypanosomatids.

**Figure 3:**
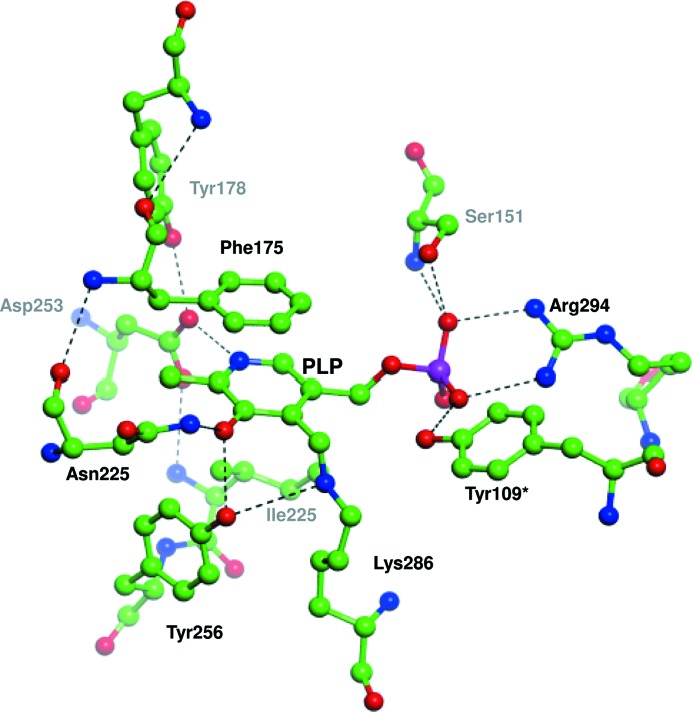
Active site with pyridoxal phosphate (PLP) bound to Lys286 and residues which stabilize PLP. C atoms, H atoms, O atoms, N atoms and phosphate are shown in green, grey, red, blue and magenta, respectively. Hydrogen bonds which participate in the stabilization of the cofactor are indicated by black dashes. Tyr109* belongs to the opposite subunit.

Reversible transamination reactions are a bi-bi ping-pong mechanism (Kirsch *et al.*, 1984 ▶), and tyrosine aminotransferases catalyze the transamination of an amino group from the donor to the covalently bound PLP to form pyridoxamine phosphate (PMP; Fig. 2 ▶). The second step of the reaction transfers the amino group to the carbonyl moiety of an amino acceptor, regenerating the prosthetic group. In both subunits of LiTAT, PLP is covalently bound to Lys286 in a cavity located at the interface between the subunits, although these cavities are not adjacent in the dimer (Fig. 3 ▶). However, residues from both subunits participate in the stabilization of PMP and their disposition is similar between LiTAT and TcTAT, although Thr184 and Tyr345 in TcTAT are replaced by Ile221 and Phe378 in LiTAT. Since the hydroxyl group of Tyr345 is oriented towards the internal aldimine, replacement by Phe378 in LiTAT may affect the reactivity of PLP with the amino donor and/or acceptor.

Unlike TcTAT (and mammalian TATs), LiTAT is not able to transaminate α-ketoglutarate using tyrosine as the amino donor (0.32 ± 0.17 U per milligram of purified protein), although it can transaminate pyruvate with high efficiency (82.3 ± 0.79 U per milligram of purified protein), consistent with the published activity of the *L. major* orthologue (Marciano *et al.*, 2009 ▶).

**Figure 4:**
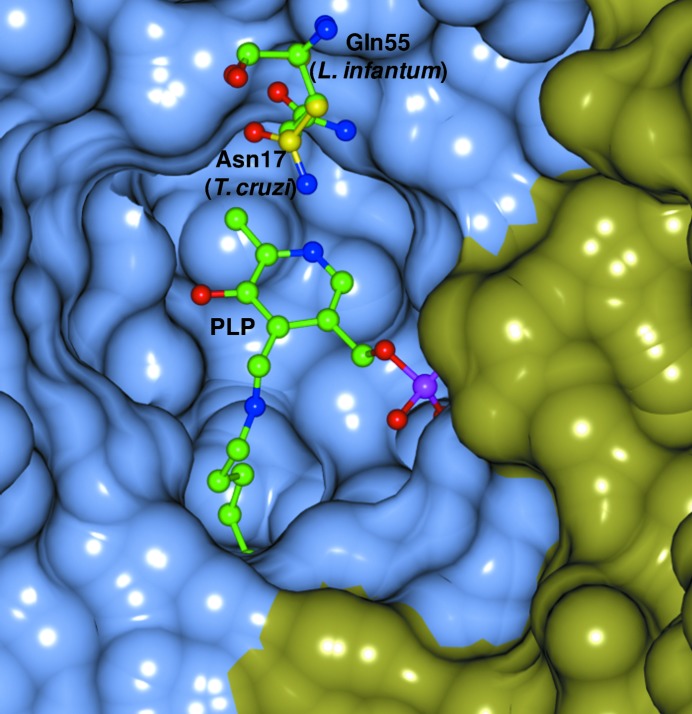
Overlay of the LiTAT structure with the TcTAT structure showing the residues involved in the recognition of the incoming substrate. The surface of chain *A* is shown in light blue and the surface of chain *B* is shown in yellow. C atoms of Gln55 and PLP of *L. infantum* are shown in green and C atoms of Asn17 of *T. cruzi* are shown in yellow. O atoms, N atoms and phosphate are shown in red, blue and magenta, respectively. The following residues were omitted for clarity: Leu55–Arg68, Thr109–Gln120, Ala138–Ser148, Phe175 and Ala311–Leu319 from chain *A* and Glu142–Ser146 and Gly319 from chain *B*.

Site-directed mutagenesis studies using rat TAT and TcTAT showed that the ability to transaminate dicarboxylic substrates was likely to be owing to the presence of residues Asn54 and Arg57 (Asn17 and Arg20 in TcTAT) helping to orient the amino acceptors towards the active centre (Sobrado *et al.*, 2003 ▶). These residues, which are conserved in most orthologues of tyrosine aminotransferases, are not present in LiTAT, where they are replaced by Gln55 and Asn58. While the side chain of Asn58 was not well resolved in our model, the side chain of Gln55 is less oriented towards the substrate-binding pocket than Asn17 in TcTAT (Fig. 4 ▶). Thus, we postulate that the difference in the specificity of *Leishmania* TATs is related to these amino-acid substitutions by altering the hydrogen-bonding inter­action with the oxoacid substrates.

## 4. Conclusion   

We have obtained a 2.35 Å resolution structure of tyrosine aminotransferase from *L. infantum*, which correlates well with those of other tyrosine aminotransferases. However, unlike other aminotransferases, *Leishmania* TAT is not able to transaminate dicarboxylic substrates and its preferred substrate is pyruvate. This can be explained by the substitution of the critical residues Asn54 and Arg57 found in other TAT orthologues by Gln55 and Asn58 in LiTAT. This difference in substrate activity, as well as the relevance of the enzyme to the life cycle of the parasite, highlight the importance of this enzyme as a potential candidate for the development of inhibitors in the near future.

## 5. Related literature   

The following references are cited in the Supporting Information for this article: Gouet *et al.* (1999 ▶, 2003 ▶).

## Supplementary Material

PDB reference: tyrosine aminotransferase, 4ix8

Supporting Information.. DOI: 10.1107/S2053230X14007845/hv5256sup1.pdf

## Figures and Tables

**Table 1:** Data-collection statistics Values in parentheses are for the highest of 20 resolution shells.

Diffraction data	
Space group	*P*3_2_21
Unit-cell parameters (Å)	*a* = *b* = 98.96, *c* = 199.26
Resolution (Å)	50–2.35 (2.41–2.35)
Mean *I*/σ(*I*)	13.8 (2.6)
*R*_merge_ †	0.069 (0.563)
*R*_mean_	0.079 (0.635)
CC_1/2_	99.8 (81.9)
Completeness (%)	99.8 (99.9)
Multiplicity	4.6 (4.7)
No. of unique reflections	47821 (3479)
Wilson *B* factor (Å^2^)	40.4
Refinement	
No. of protein atoms	5859
No. of waters	274
No. of other atoms	50
*R*_work_ ‡	0.173 (0.261)
*R*_free_ §	0.206 (0.325)
R.m.s.d., bonds (Å)	0.012
R.m.s.d., angles (°)	1.35
Ramachandran, favoured	745 [97.9%]
Ramachandran, outliers	None
Average *B* factor (Å^2^)	
Overall	55.8
Protein	56.3
Solvent	43.5
*MolProbity* clashscore¶	1.31 [100th percentile]
*MolProbity* score¶	0.88 [100th percentile]
PDB entry	4ix8

† *R*_merge_ = 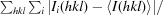 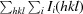.

‡ *R*_work_ = 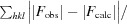 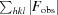.

§ The free *R* factor was calculated with an equivalent equation to *R*_work_ using 5% of the reflections that were omitted from the refinement.

¶ Chen *et al.* (2010 ▶).

## Footnotes

1 Supporting information has been deposited in the IUCr electronic archive (Reference: HV5256).
