# Supplementary material for: Structure of tyrosine aminotransferase from Leishmania infantum
Source: Acta Crystallogr F Struct Biol Commun. 2014 Apr 25;70(Pt 5):583–7. doi: 10.1107/S2053230X14007845 (PMC4014323; doi:10.1107/S2053230X14007845)
Supplement: Supplementary file 1 [file f-70-00583-sup1.pdf]

Acta Crystallographica Section F

Volume 70 (2014)

Supporting information for article:

Structure of tyrosine aminotransferase from *Leishmania infantum*

M. A. Moreno, A. Abramov, J. Abendroth, A. Alonso, S. Zhang, P. J. Alcolea, T. Edwards, D. Lorimer, P. J. Myler and V. Larraga

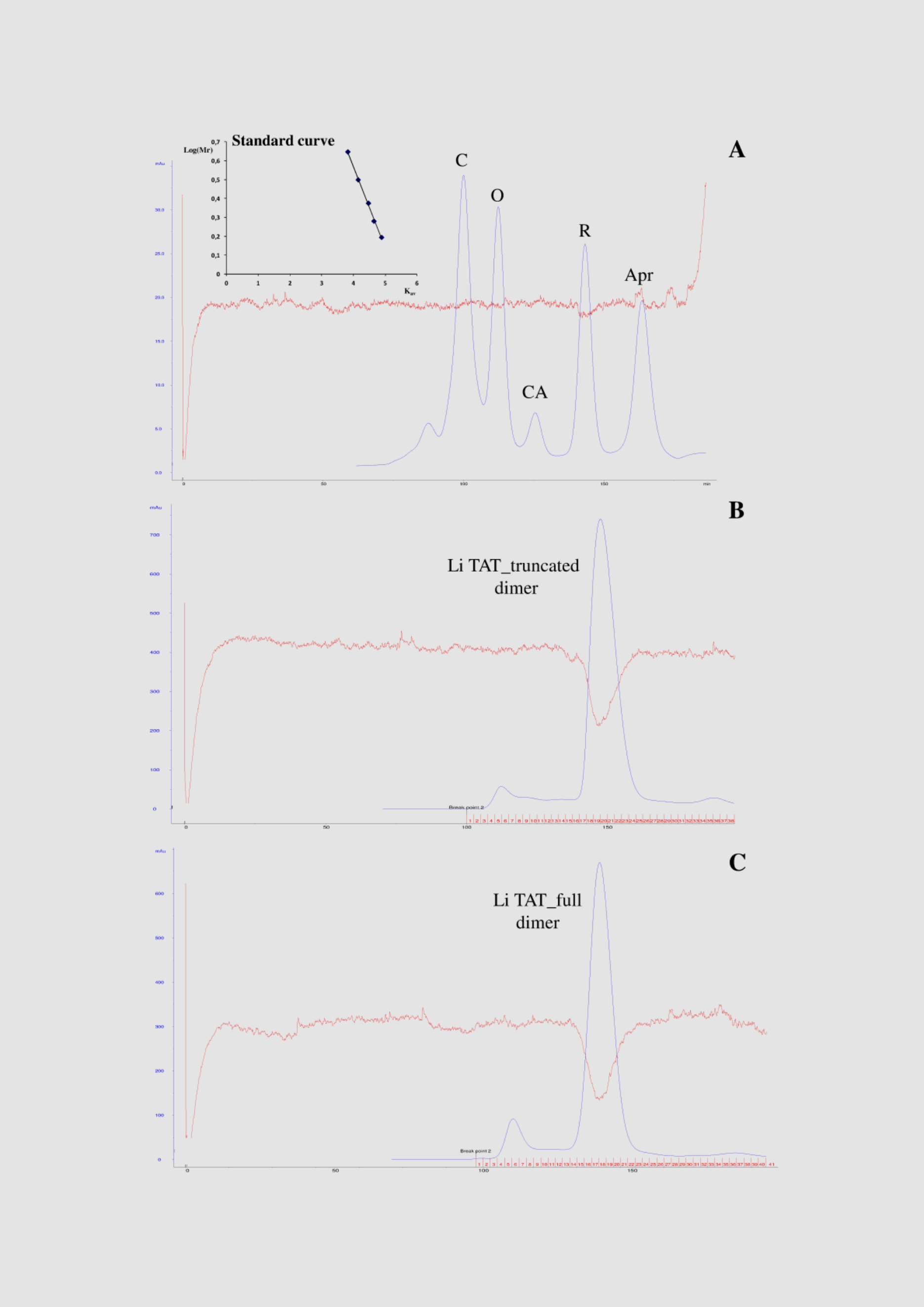

Figure S1 LiTAT_full and LiTAT_truncated form a dimer in solution. A. A calibration curve was obtained based on the gel base distribution coefficient ($K_{av)}$ and the logarithmic of the molecular weight (Mr) of five standard proteins included in the Low Molecular Weight Kit. O: Ovoalbumin, C: Conalbumin, CA: Carbonic anhidrase, R: Ribonuclease A, Apr: Aprotinin. **B.** Gel filtration chromatogram corresponding to LiTAT_truncated purification. The estimated K_{av} value was 0,136 and the estimated Mr was 100,42 kDa. **C.** Gel filtration chromatogram corresponding to LiTAT_full purification. The estimated K_{av} value was 0,1095 and the estimated Mr was 115,62 kDa.

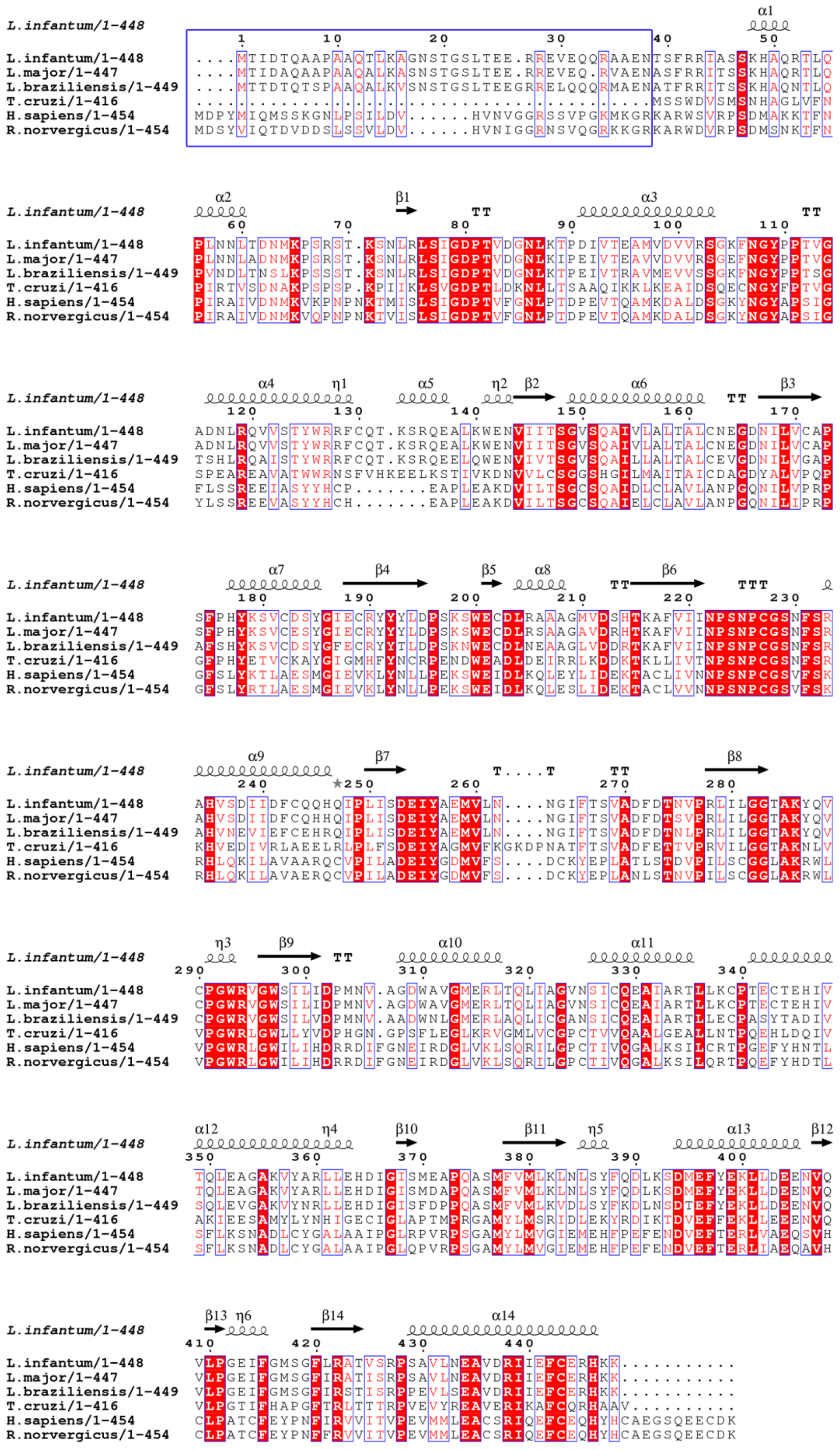
L.infantum/1-448
L.infantum/1-448 ....MTIDTQAAPAAQTLKAGNSTGSLTEE.RREVEQQRAAENTSFRRIASSKHAQRTLQ
L.major/1-447 ....MTIDAQAAPAAQALKASNSTGSLTEE.RREVEQ.RVAENASFRRITSSKHAQRTLQ
L.braziliensis/1-449 ....MTTDTQTSPAAQALKVSNSTGSLTEEGRRELQQQRMAENATFRRITSSKHAQNTLQ
T.cruzi/1-416 ....MSSWDVSMSNHAGLVFN
H.sapiens/1-454 MDPYMIQMSSKGNLPSILDV......HVNVGGRSSVPGKMKGRKARWSVRPSDMAKKTFN
R.norvergicus/1-454 MDSYVIQTDVDDSLSSVLDV......HVNIGGRNSVQGRKKGRKARWDVRPSDMSNKTFN
L.infantum/1-448 PLNNLTDNMKPSRST.KSNLRLSIGDPTVDGNLKTPDIVTEAMVDVVRSGKFNGYPPTVG
L.major/1-447 PLNNLADNMKPSRST.KSNLRLSIGDPTVDGNLKIPEIVTEAVVDVVRSGEFNGYPPTVG
L.braziliensis/1-449 PVNDLTNSLKPSSST.KSNLRLSIGDPTVDGNLKTPEIVTRAVMEVVSSGKFNGYPPTSG
T.cruzi/1-416 PIRTVSDNAKPSPSP.KPIIKLSVGDPTLDKNLLTSAAQIKKLKEAIDSQECNGYFPTVG
H.sapiens/1-454 PIRAIVDNMKVKPNPNKTMISLSIGDPTVFGNLPTDPEVTQAMKDALDSGKYNGYAPSIG
R.norvergicus/1-454 PIRAIVDNMKVQPNPNKTVISLSIGDPTVFGNLPTDPEVTQAMKDALDSGKYNGYAPSIG
L.infantum/1-448 ADNLRQVVSTYWRRFCQT.KSRQEALKWENVIITSGVSQAIVLALTALCNEGDNILVCAP
L.major/1-447 ADNLRQVVSTYWRRFCQT.KSRQEALKWENVIITSGVSQAIVLALTALCNEGDNILVCAP
L.braziliensis/1-449 TSHLRQAISTYWRRFCQT.KSRQEELQWENVIVTSGVSQAILLALTALCEVGDNILVGAP
T.cruzi/1-416 SPEAREAVATWWRNSFVHKEELKSTIVKDNVVLCSGGSHGILMAITAICDAGDYALVPQP
H.sapiens/1-454 FLSSREEIASYYHCP.......EAPLEAKDVILTSGCSQAIDLCLAVLANPGQNILVPRP
R.norvergicus/1-454 YLSSREEVASYYHCH.......EAPLEAKDVILTSGCSQAIELCLAVLANPGQNILIPRP
L.infantum/1-448 SFPHYKSVCDSYGIECRYYYLDPSKSWECDLRAAAGMVDSHTKAFVIINPSNPCGSNFSR
L.major/1-447 SFPHYKSVCESYGIECRYYYLDPSKSWECDLRSAAGAVDRHTKAFVIINPSNPCGSNFSR
L.braziliensis/1-449 AFSHYKSVCDSYGFECRYYTLDPSKNWECDLNEAAGLVDDRTKAFVIINPSNPCGSNFSR
T.cruzi/1-416 GFPHYETVCKAYGIGMHFYNCRPENDWEADLDEIRRLKDDKTKLLIVTNPSNPCGSNFSR
H.sapiens/1-454 GFSLYKTLAESMGIEVKLYNLLPEKSWEIDLKQLEYLIDEKTACLIVNNPSNPCGSVFSK
R.norvergicus/1-454 GFSLYRTLAESMGIEVKLYNLLPEKSWEIDLKQLESLIDEKTACLVVNNPSNPCGSVFSK
L.infantum/1-448 AHVSDIIDFCQQHQIPLISDEIYAEMVLN....NGIFTSVADFDTNVPRLILGGTAKYQV
L.major/1-447 AHVSDIIDFCQHHQIPLISDEIYAEMVLN....NGIFTSVADFDTSVPRLILGGTAKYQV
L.braziliensis/1-449 AHVNEVIEFCEHRQIPLISDEIYAEMVLN....NGIFTSVADFDTNLPRLILGGTAKYQV
T.cruzi/1-416 KHVEDIVRLAEELRLPLFSDEIYAGMVFKGKDPNATFTSVADFETTVPRVILGGTAKNLV
H.sapiens/1-454 RHLQKILAVAARQCVPILADEIYGDMVFS....DCKYEPLATLSTDVPILSCGGLAKRWL
R.norvergicus/1-454 RHLQKILAVAERQCVPILADEIYGDMVFS....DCKYEPLANLSTNVPILSCGGLAKRWL
L.infantum/1-448 CPGWRVGWSILIDPMNV.AGDWAVGMERLTQLIAGVNSICQEAIARTLLKCPTECTEHIV
L.major/1-447 CPGWRVGWSILIDPMNV.AGDWAVGMERLTQLIAGVNSICQEAIARTLLKCPTECTEHIV
L.braziliensis/1-449 CPGWRVGWSILVDPMNV.AADWNLGMERLAQLICGANSICQEAIARTLLECPASYTADIV
T.cruzi/1-416 VPGWRLGWLLYVDPHGN.GPSFLEGLKRVGMLVCGPCTVVQAALGEALLNTPQEHLDQIV
H.sapiens/1-454 VPGWRLGWILIHDRRDIFGNEIRDGLVKLSQRILGPCTIVQGALKSILCRTPGEFYHNTL
R.norvergicus/1-454 VPGWRLGWILIHDRRDIFGNEIRDGLVKLSQRILGPCTIVQGALKSILQRTPQEFYHDTL
L.infantum/1-448 TQLEAGAKVYARLLEHDIGISMEAPQASMFVMLKLNLSYFQDLKSDMEFYEKLLDEENVQ
L.major/1-447 TQLEAGAKVYARLLEHDIGISMDAPQASMFVMLKLNLSYFQDLKSDMEFYEKLLDEENVQ
L.braziliensis/1-449 SQLEVGAKVYNRLLEHDIGISFDPPQASMFVMLKVDLSYFKDLNSDTEFYEKLLDEENVQ
T.cruzi/1-416 AKIEESAMYLYNHIGECIGLAPTMPRGAMYLMSRIDLEKYRDIKTDVEFFEKLLEEENVQ
H.sapiens/1-454 SFLKSNADLCYGALAAIPGLRPVRPSGAMYLMVGIEMEHFPEFENDVEFTERLVAEQSVH
R.norvergicus/1-454 SFLKSNADLCYGALAAIPGLQPVRPSGAMYLMVGIEMEHFPEFENDVEFTERLIAEQAVH
L.infantum/1-448 VLPGEIFGMSGFLRATVSRPSAVLNEAVDRIIEFCERHKK..........
L.major/1-447 VLPGEIFGMSGFIRATISRPSAVLNEAVDRIIEFCERHKK..........
L.braziliensis/1-449 VLPGEIFGMSGFIRSTISRPPEVLSEAVDRIIEFCERHKK..........
T.cruzi/1-416 VLPGTIFHAPGFTRLTTTRPVEVYREAVERIKAFCQRHAAV.........
H.sapiens/1-454 CLPATCFEYPNFIRVVITVPEVMMLEACSRIQEFCEQHYHCAEGSQEECDK
R.norvergicus/1-454 CLPATCFEYPNFFRVVITVPEVMMLEACSRIQEFCEQHYHCAEGSQEECDK

Figure S2 Multiple sequence alignment of the *L. infantum*, *L. major*, *L. braziliensis*, *T. cruzi*, *Homo sapiens* and *Rattus norvergicus* tyrosine aminotransferases. The figure was generated with ESPript (Gouet *et al.*, 1999, Gouet *et al.*, 2003), with conserved regions indicated by boxes. Residues identical in all sequences are highlighted red and conservative replacements indicated by red text. The N-terminal domain absent from TcTAT is outlined by the blue box. Secondary structure features obtained from the LiTAT structure are shown above the sequence, with alpha- or 3_{10}- helices, beta-sheets and hydrogen bonded turns indicated by α, η, β, and T, respectively.
